# Integrative analysis of plasma metabolomics and proteomics reveals the metabolic landscape of breast cancer

**DOI:** 10.1186/s40170-022-00289-6

**Published:** 2022-08-17

**Authors:** Rui An, Haitao Yu, Yanzhong Wang, Jie Lu, Yuzhen Gao, Xinyou Xie, Jun Zhang

**Affiliations:** 1 Department of Clinical Laboratory, Sir Run Run Shaw Hospital, Zhejiang University School of Medicine, 3 East Qingchun Road, Hangzhou, Zhejiang 310016 People’s Republic of China; 2 Key Laboratory of Precision Medicine in Diagnosis and Monitoring Research of Zhejiang Province, 3 East Qingchun Road, Hangzhou, Zhejiang 310016 People’s Republic of China

**Keywords:** Breast neoplasms, Plasma, Metabolomics, Proteomics, Machine learning

## Abstract

**Background:**

Breast cancer (BC) is the most commonly diagnosed cancer. Currently, mammography and breast ultrasonography are the main clinical screening methods for BC. Our study aimed to reveal the specific metabolic profiles of BC patients and explore the specific metabolic signatures in human plasma for BC diagnosis.

**Methods:**

This study enrolled 216 participants, including BC patients, benign patients, and healthy controls (HC) and formed two cohorts, one training cohort and one testing cohort. Plasma samples were collected from each participant and subjected to perform nontargeted metabolomics and proteomics. The metabolic signatures for BC diagnosis were identified through machine learning.

**Results:**

Metabolomics analysis revealed that BC patients showed a significant change of metabolic profiles compared to HC individuals. The alanine, aspartate and glutamate pathways, glutamine and glutamate metabolic pathways, and arginine biosynthesis pathways were the critical biological metabolic pathways in BC. Proteomics identified 29 upregulated and 2 downregulated proteins in BC. Our integrative analysis found that aspartate aminotransferase (GOT1), l-lactate dehydrogenase B chain (LDHB), glutathione synthetase (GSS), and glutathione peroxidase 3 (GPX3) were closely involved in these metabolic pathways. Support vector machine (SVM) demonstrated a predictive model with 47 metabolites, and this model achieved a high accuracy in BC prediction (AUC = 1). Besides, this panel of metabolites also showed a fairly high predictive power in the testing cohort between BC vs HC (AUC = 0.794), and benign vs HC (AUC = 0.879).

**Conclusions:**

This study uncovered specific changes in the metabolic and proteomic profiling of breast cancer patients and identified a panel of 47 plasma metabolites, including sphingomyelins, glutamate, and cysteine could be potential diagnostic biomarkers for breast cancer.

**Supplementary Information:**

The online version contains supplementary material available at 10.1186/s40170-022-00289-6.

## Background

Breast cancer (BC) is the first major malignancy seriously threatened the health of females worldwide. According to the latest statistics, female BC has already exceeded lung cancer to become the most commonly diagnosed cancer with an estimated 2.3 million (11.7%) new cases in 2020 [1]. In China, BC is also the most frequently occurring malignancy among females with approximately 304,000 (17.1%) new BC cases reported in 2015 [2]. In recent years, along with societal development, a 20- to 40-year-old young BC patients have significantly increased, and BC onset shows a trend toward younger age. Among new diagnosed BC cases, approximately 3 to 10% patients were accompanied with distant metastasis. Approximately 30% of early-stage patients can progress to advanced BC [3]. The prognosis of BC was found to be closely related to the developmental stage of the disease. Specifically, the overall 5-year survival rate of patients with BC is 89.9%, in which the 5-year survival rate is close to 100% for carcinoma in situ, and 85.5% for early-stage invasive carcinoma, but only 25% for distant metastasis [4]. Therefore, improving the early diagnosis rate of BC and giving timely and effective treatment is crucial to improve the survival rate of BC patients.

Many factors are closely related to the occurrence of BC, including aging, family history, reproductive factors, estrogen, and life styles. Additionally, mutations and abnormal amplifications of many genes, such as breast cancer associated genes 1 and 2 (BRCA1/2), human epidermal growth factor receptor 2 (HER2), and epidermal growth factor receptor (EGFR) also play an important role in the occurrence and development of BC [5, 6]. At present, the pathogenesis of BC has not been fully demonstrated. In addition to genetic factors, environmental factors are also involved in the occurrence and development of the disease. Lécuyer et al. found high levels of glucose, creatinine, glutamine, arginine, lysine, and valine to be closely associated with a higher risk of BC [7]. Another study reported that a higher level of glutamine/isoglutamine, valine/norvaline, tryptophan, phenylalanine, γ-glutamyl-threonine, 5-aminovaleric acid, or 5-aminovaleric acid was related to an increased risk of BC [8]. These findings illustrated that metabolites may play an important role in the occurrence and development of BC. Currently, guidelines have recommended that imaging be used to screen for BC, including mammography, breast ultrasonography, and breast MRI. However, the sensitivity of these methods is not high; therefore, additional scrutiny by breast tissue biopsy is typically needed to achieve a more accurate diagnosis [9, 10]. Serum tumor markers (TM) for clinical screening of BC, such as carcinoembryonic antigen (CEA) and carbohydrate antigen 15-3 (CA15-3), have been widely used [11]. However, both the two TMs have poor sensitivity and specificity. Therefore, it is crucial to identify more sensitive biomarkers, along with understanding the mechanisms by which these biomarkers promote the onset and development of BC. One answer lies in the use of metabolomics technology. Metabolomics performs a quantitative analysis of all metabolites in an organism and looks for the relative relationship between metabolites and physiopathological changes [12]. Unlike genomics and proteomics, the focus of metabolomics is on the downstream products of genes and proteins, allowing more accurate identification of disease-associated changes that have occurred, rather than predictions [13].

At present, metabolomics technology has successfully been used to identify biomarkers in various tumors, such as lung cancer [14], pancreatic ductal adenocarcinoma [15], prostate cancer [16], bladder cancer [17], and breast cancer [18]. Many metabolomics studies have focused on BC. These studies have found specific biomarkers for diagnosis and therapeutic response prediction in BC according to the metabolomics analysis based on human serum, tissues, or urine [19–21]. However, some studies only reported on a small sample size, while the results of others were inconsistent. No studies, to the best of our knowledge, have systematically explored the possible mechanisms underlying key metabolic changes in BC. Therefore, we herein evaluated the plasma metabolomics of BC patients and healthy individuals to identify cancer-associated changes in metabolites and metabolic pathways. And identified particular metabolite signatures through machine learning, which can used for BC early diagnosis.

## Methods

### Study design and participants enrollment

As shown in Supplementary Fig. S1, two cohorts of participants were recruited from Sir Run Run Shaw Hospital, Zhejiang University School of Medicine, to perform the landscape of plasma metabolomics and proteomics in BC patients, including one training cohort and one testing cohort. First, we measured metabolite profiles across the two cohorts. Second, 9 BC patients and 9 HC individuals were randomly selected from the training cohort to conduct the proteomics analysis. We then performed an integrative analysis of metabolomics and proteomics data. Finally, we used the differential metabolites in the training cohort to train a machine learning models and followed up by testing in the testing cohort.

The training cohort comprised 75 BC patients, 30 benign patients, and 20 healthy controls (HC) enrolled from January 2018 to December 2018. The testing cohort comprised 32 BC patients, 30 benign patients, and 29 HC individuals recruited from October 2019 to June 2020. Demographic and clinical data of the participants, including age, sex, body mass index (BMI), menstrual history, and family history were collected. For BC patients, special clinicopathologic features, including tumor type at the molecular level, tumor stage, CA15-3, and CEA, were also collected. Then, in the training cohort, a TMT-labeled quantitative proteomics analysis was performed for 9 randomly selected BC patients and 9 HC individuals. The study was carried out in accordance with the Declaration of Helsinki. All participants were properly familiarized with our study and provided a signed informed consent. The study protocol was approved by the Ethics Committee of Sir Run Run Shaw Hospital, Zhejiang University School of Medicine (20180601-006). Inclusion criteria required that BC patients (1) be 18 to 79 years old and (2) be histopathologically confirmed with BC with no other malignancies. Further, (3) BC patients should not have undergone previous surgery, chemotherapy, or radiotherapy before enrollment. The inclusion criteria for HC participants required that they (1) be 18 to 79 years old and (2) have no history of tumor or other breast diseases. Other criteria excluded any participant with (1) systemic chronic disease, such as cardiovascular diseases, hypertension, and diabetes; (2) metabolism-related disease, such as phenylketonuria and hepatic encephalopathy; (3) mental illness; and (4) females in menstruation, pregnancy, or lactation.

### Plasma collection and preservation

Plasma samples for all participants were collected in the morning after overnight fasting (8–14h). Blood samples were collected using an EDTA anticoagulant tube and left at room temperature for 30 min. Then, the samples were centrifuged at 3000 rpm for 10 min. Supernatant (plasma) was collected in 1.5ml frozen tubes and stored at −80°C until further analyses.

### Preparation of plasma samples for metabolomics

Samples were prepared using the automated MicroLab STAR® system from the Hamilton Company. Several recovery standards were added prior to the first step in the extraction process for quality control (QC) purposes. To remove protein, small molecules bound to protein, or trapped in the precipitated protein matrix, were dissociated, and chemically diverse metabolites were recovered. Proteins were precipitated with methanol under vigorous shaking for 2 min (Glen Mills GenoGrinder 2000, USA), followed by centrifugation. The samples were placed briefly on a TurboVap® (Zymark) to remove the organic solvent. The sample extracts were stored overnight under nitrogen before preparation for analysis. Small aliquots of each plasma sample were pooled to create QC samples, which were then injected periodically throughout the platform run.

### Ultrahigh performance liquid chromatography-tandem mass spectroscopy analysis (UPLC-MS/MS analysis)

Metabolomics was performed by Metabolon Inc. (DIAN-09-19VW) (Durham, NC, USA), using Waters ACQUITY ultra-performance liquid chromatography (UPLC) and a Thermo Scientific Q-Exactive high-resolution mass spectrometer which was interfaced with a heated electrospray ionization (HESI-II) source and Orbitrap mass analyzer. All samples in the discovery cohort were detected within the same batch, and all samples in the testing cohort were also detected within the same batch. Extracts were dried and reconstituted in solvent compatible with each of the methods. Hydrophilic compounds were analyzed using positively charged ions electrospray ionisation under acidic conditions. Specifically, the extracts underwent gradient elution from a C18 column (Waters UPLC BEH C18-2.1×100 mm, 1.7 μm), using water and methanol that consisted of 0.05% perfluoropentanoic acid (PFPeA) and 0.1% formic acid (FA). Hydrophobic compounds were also analyzed using positively charged ion electrospray ionization under acidic conditions with the same C18 column, as noted above, using methanol, acetonitrile, water, 0.05% PFPeA, and 0.01% FA, operated at an overall higher organic content. Basic extracts were analyzed using basic negative ion conditions, the extracts underwent gradient elution from a dedicated C18 column using methanol, water, and 6.5mM ammonium bicarbonate at pH 8. Then, a negative ionization, following the elution from a hydrophilic interaction liquid chromatography (HILIC) column (Waters UPLC BEH Amide 2.1×150 mm, 1.7 μm), was used for extract gradient-elution, using a gradient consisting of water and acetonitrile with a 10-mM ammonium formate at pH 10.8. MS analysis alternated between MS and data-dependent MSn scans using dynamic exclusion. The scan range varied slightly between methods, but covered 70–1000 m/z.

### Metabolomics analysis

Raw data extraction, peak identification, and QC processing were performed through Metabolon’s hardware and software. Compounds were identified by comparison with library entries of purified standards or recurrent unknown entities. The library contained retention time/index (RI), mass to charge ratio (m/z), and chromatographic data, including MS/MS spectral data, on all molecules present in the library. Biochemical identification was based on three other criteria, as follows: (1) retention index within a narrow RI window of the proposed identification, (2) accurate mass match to the library ± 10 ppm, and (3) MS/MS forward and reverse scores between the experimental data and authentic standards. A variety of curation procedures were carried out to ensure that a high-quality data set was made available for statistical analysis and data interpretation.

Peaks were quantified using the area under the curve, and the missing values were replaced by LoDs (1/5 of the minimum positive value of the variable). Then, normalization and data scaling were completed by MetaboAnalyst 5.0 (https://www.metaboanalyst.ca/), the parameter used for normalization was “Normalization by sum,” and the parameter used for Data scaling was “auto scaling (mean-centered and divided by the standard deviation of each variable)”. In the univariate analysis phase, fold change threshold (FC) of each metabolite was calculated, and Student’s *t* test or Mann–Whitney *U* test was applied to measure the differences in metabolites between groups. In the multivariate analysis stage, orthogonal projections to latent structures discriminant analysis (OPLS-DA) was used to identify important metabolites selected according to variable importance in the projection (VIP > 1.0). Metabolites were considered significantly altered based on the following criteria: FC > 1.2 or < 5/6, VIP >1, and *P* < 0.05. Metabolites meeting these criteria were subjected to KEGG pathway analysis to search for specific metabolic pathways closely associated with BC.

### Preparation of plasma samples for proteomics

Before the proteomics experiment, plasma samples were centrifuged at 15,000g for 15 min. Then, the supernatant was filtered with a 0.22-μM filter. The top 14 high-abundance proteins (albumin, IgG, antitrypsin, IgA, transferrin, haptoglobin, fibrinogen, alpha2-macroglobulin, alpha1-acid glycoprotein, IgM, apolipoprotein AI, apolipoprotein AII, complement C3, and transthyretin) were removed using immunoaffinity chromatography (IAC). Then, the low-abundance proteins underwent reduction, alkylation, and specific trypsin lysis. The digested peptides were subjected to 10-plex TMT-labeling, followed by freeze-drying to a powdered form. The freeze-dried samples were reconstituted with 0.1% trifluoroacetic acid (TFA) and filtered using a 0.22-μM filter. The filtered samples were subjected to high-pH RP-HPLC separation, and the chromatographically separated components were collected. Mobile phase A was 5% NH_3_H_2_O+95% H_2_O, and mobile phase B was acetonitrile+5% NH_3_H_2_O+5% H_2_O. A total of 25 components were used to perform nano-LC-MS/MS analysis.

### Nano-LC-MS/MS analysis

Twenty-five chromatographic components obtained from high-pH RP-HPLC were analyzed using a Q Exactive HF-X high-resolution mass spectrometer that was coupled with an Easy nLC-1200 liquid chromatography system. Mobile phase-A was 0.1% formic acid in water, and mobile phase-B was 0.1% formic acid in acetonitrile (80% acetonitrile and 20% water). The column consisted of an enrichment and analytical column and was equilibrated with 100% mobile phase-A. Samples were loaded onto an enrichment column (100μm_ID×4cmL, C18, 3μm, 100A) by an autosampler and separated using an analytical column (75 μm_ID×25cmL, C18, 3 μm, 100A). Then, mass spectrometry was performed with a Q Exactive HF-X mass spectrometer. The detection mode was positive ions and parent ion scan range of 350–1800 M/Z with a primary MS resolution of 120,000 at 200 m/Z, and AGC (automatic gain control) target was set to 3×10^6^ ions, the maximum injection time to 50 ms and dynamic exclusion time of 40s. The mass charge ratios of peptides and peptide fragments were collected according to the data-dependent acquisition (DDA) method whereby 20 secondary profiles (MS/MS, MS2 scan) were acquired after each full scan (full scan, primary mass spectrometry).

### Proteomics data analysis

Acquired spectra were searched against the complete proteome set of *Homo sapiens* from SwissProt (released version 2020_05). Database search parameters were set as follows: maximum of two missed cleavage sites permitted for trypsin digestion, 10-ppm precursor mass tolerance, 0.02-Da fragment mass tolerance, cysteine carbamidomethylation (CAM) modification (+57.021 Da) as a static modification, and oxidation modification for methionine (+15.995 Da) as a dynamic modification. LC-MS/MS data were processed and analyzed using Proteome Discoverer, version 2.4 (ThermoFisher Scientific). All searches were filtered to a <1% false discovery rate (FDR).

The peak intensity of TMT-labeled peptide was normalized, and the upregulated proteins (BC/HC > 1.25) and downregulated proteins (BC/HC < 0.80) were screened according to a fold change of 1.25. Proteins identified and quantified in the experiment were compared with the whole set of proteins in the standard database, followed by Gene Ontology (GO) analysis. Then, the direct and indirect relationships between the differential proteins using String (https://string-db.org/), and Cytoscape, version: 3.2.1, was used to generate and analyze the interaction network.

### Integration of metabolomics and proteomics

First, the correlation analysis between metabolomics and proteomics was performed using metabolites and proteins that were significantly different between BC and HC groups. Then, the metabolites and proteins with significant differences between groups were subjected to Joint Pathway Analysis using MetaboAnalyst 5.0 (https://www.metaboanalyst.ca/).

### Machine learning for the prediction of breast cancer

For BC vs. non-BC (benign + HC), important metabolites were first selected based on the following criteria: *P* < 0.05, VIP > 0.5, and AUC > 0.6. Then, we performed Lasso regression 10-fold cross validation and Random Forest by R packages “glmnet” and “Boruta” to select potential metabolites used to train a disease prediction model. Based on the above candidates, the support vector machine (SVM) and random forest (RF) models were applied using the R packages “e1071” and “randomForest,” and the receiver operating characteristic (ROC) curves and area under the curve (AUC) were calculated by “pROC” from R package. Then, the SVM model was applied in the testing cohort, and the AUC was calculated to evaluate the diagnostic efficacy of the model.

### Statistical analysis

All statistical analyses were performed by SPSS 26.0 (Statistical Product and Service Solutions, IBM, USA) and R, version 3.5.2 (R Foundation for Statistical Computing, Austria). Numerical variable data were expressed as mean ± standard deviation (mean ± SD). Comparisons between numerical variables were performed using Student’s *t* test, Mann–Whitney *U* test, or one-way ANOVA analysis. Comparisons between categorical variables were performed by chi-square test. Correlation analysis between different variables was performed by Spearman’s rank correlation analysis. *P* < 0.05 was considered as statistical significance, and the original *P* values of multiple tests were adjusted by FDR (Benjamin–Hochberg).

## Results

### Demographic characteristics of the participants

Herein, metabolomics and proteomics analyses of plasma samples were performed to investigate the differences in metabolites and proteins among BC, benign, and HC individuals and to identify diagnostic markers of BC patients. In the training cohort, a total of 75 BC patients (all female), 30 benign patients (all female), and 20 HC (all female) were assessed for metabolomic analysis. BC patients were older than benign patients and HC individuals (52.03 ± 10.62 vs*.* 43.60 ± 11.93 vs*.* 44.68 ± 13.20, respectively, *P* = 0.001). There was no significant difference in the population of post-menopausal woman among BC, benign, and HC groups (40%, 26.7%, 35%, respectively, *P* > 0.05). Then, 9 BC patients and 9 HC individuals were randomly selected from the training cohort to conduct the proteomics analysis. In the testing cohort, 32 BC patients (all females), 30 benign patients (all females), and 29 HC individuals (all females) were analyzed. In this cohort, BC patients were also older than participants in the other two groups (53.81 ± 11.15 vs*.* 39.60 ± 11.67 vs*.* 37.97 ± 8.60, respectively, *P* < 0.001) and also had a higher proportion of post-menopausal women compared to the benign group (37.5 vs*.* 10%, respectively, *P* < 0.001). The tumor markers CEA and CA15-3 for BC, CEA > 5ng/ml and CA15-3 > 25 U/ml, were each considered as BC-positive, respectively. And the BC group had a higher BC-positive rate compared to benign and HC groups according to CA15-3 and CEA (18.8% and 6.3%, respectively). No significant difference in BMI was observed among the three groups both in training and testing cohorts. The detailed demographic and clinical characteristics of the training and testing cohorts were shown in Table 1 and Supplementary Table 1.

**Table 1:** Demographic characteristics of training cohort participants

Parameters	Healthy controls	Benign patients	BC patients	*P* value
Number of samples	20	30	75	
Age (years, mean ± SD)	44.68 ± 13.20	43.60 ± 11.93	52.03 ± 10.62	0.001
Gender (female)	20	30	75	-
BMI (mean±SD, kg/m^2^)	22.47 ± 2.81	22.49 ± 2.29	23.02 ± 3.28	0.634
Tumor molecular type	NA	NA		-
Luminal A			19 (25.3%)	
Luminal B			34 (45.3%)	
HER-2^+^			14 (18.7%)	
Basal-like			8 (10.7%)	
Menopause				0.435
Post-menopausal women	7 (35%)	8 (26.7%)	30 (40%)	
Pre-menopausal women	13 (65%)	22 (73.3%)	45 (60%)	
Tumor stage	NA	NA		-
I			31 (41.33%)	
II			33 (44%)	
III			11 (14.67%)	

Continuous variables (age and BMI) were tested with one-way ANOVA analysis. Dichotomous data (menopause) was tested with chi-square test, assigned “post-menopausal women” = 1 and “pre-menopausal women” = 2. “*P* < 0.05” was considered as a statistical significance

*Abbreviations*: *BC* breast cancer, *BMI* body mass index, *NA* not applicable

### Plasma metabolic profiles

**Fig. 1:**
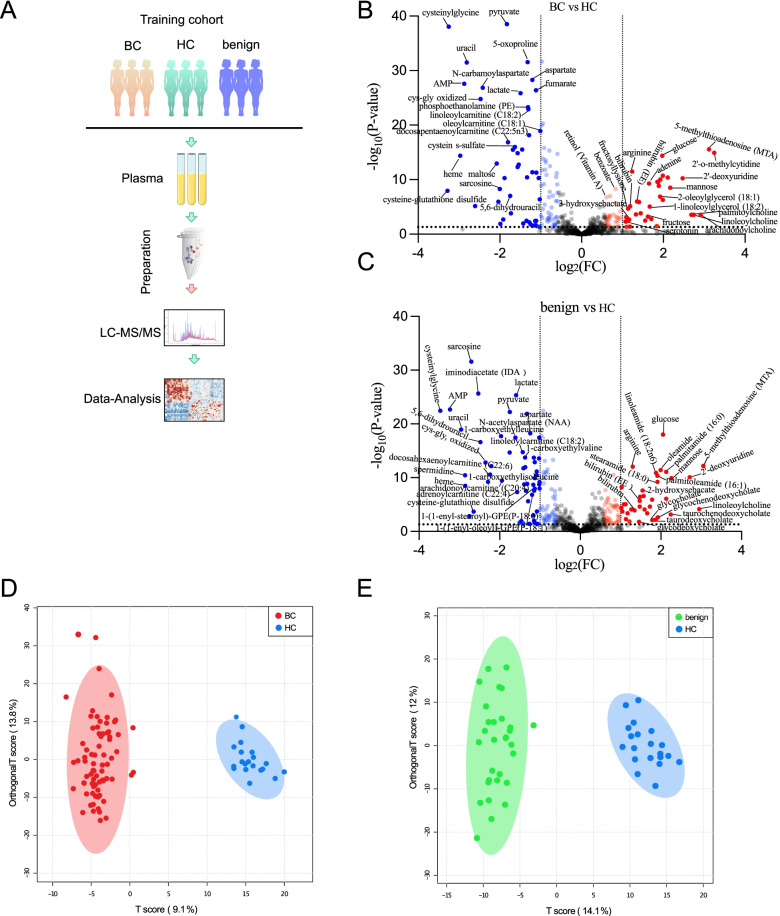
Metabolomics showed differences among groups of BC, benign, and HC. **A** Working pipeline for metabolomic analysis. **B**, **C** Volcano plot showing the metabolites that were significantly different between BC and HC groups and benign and HC groups, respectively. Each point represents a metabolite, red: upregulated metabolites, blue: downregulated metabolites. **D**, **E** Score plots of OPLS-DA models showing the separation between BC and HC groups and benign and HC groups, respectively. Each point represents a sample, red: BC patients, green: benign patients, blue: HC controls

To assess the specific metabolic profiles of BC, we performed an untargeted metabolomics analysis of paired plasma samples based on UPLC-MS/MS in the training cohort, finally identifying and quantifying 917 metabolites in the three groups (Fig. 1A). After removing the metabolites with more than 20% missing values, 750 metabolites were further analyzed. Then, after normalizing the raw data and assigning missing values with LoDs, we firstly calculated the FC and *P* values of each metabolite in each pairwise comparisons (BC vs. HC, benign vs. HC, and BC vs. benign). Next, we applied the volcano plots of pairwise comparisons within BC, benign, and HC to show the expression of metabolites. For BC vs. HC (Fig. 1B), the most abundant metabolites in BC mainly included the primary bile acid metabolism compounds (taurocholate, taurochenodeoxycholate, glycocholate, allantoin); secondary bile acid metabolism compounds (taurodeoxycholate, glycodeoxycholate, ursodeoxycholate); fructose, mannose, and galactose metabolism compounds (mannose and fructose); tyrosine metabolism compounds (tyramine O-sulfate, *N*-formylphenylalanine, dopamine 4-sulfate); and glycerolipid metabolism compounds (glycerol and glycerol 3-phosphate). The HC group had a higher level of fatty acid metabolism (acyl carnitine) compounds (oleoylcarnitine (C18:1) and docosapentaenoylcarnitine (C22:5n3)); glutathione metabolism compounds (cysteinylglycine, cys-gly, oxidized, cysteine-glutathione disulfide); glycolysis, gluconeogenesis, and pyruvate metabolism compounds (pyruvate and lactate); and citrate cycle (TCA cycle) pathway metabolism compounds (fumarate, malate, succinate), as well as urea cycle, arginine, and proline metabolism compounds (*N*-methylproline and ornithine). For benign vs*.* HC (Fig. 1C), the most abundant metabolites in benign patients included fatty acid, amide metabolism compounds (oleamide, palitamide (16:0), linoleamide (18:2n6), and palmitoleamide (16:1)); primary bile acid metabolism compounds (glycocholate, glycochenodeoxycholate); secondary bile acid metabolism compounds (taurodeoxycholate, glycodeoxycholate); and fatty acid, dicarboxylate metabolism compounds (2-hydroxysebacate, octadecadienedioate (C18:2-DC)). Metabolites with higher level in HC were similar to the above. However, no obvious difference between BC and benign was noted (Supplementary Fig. S2A).

**Fig. 2:**
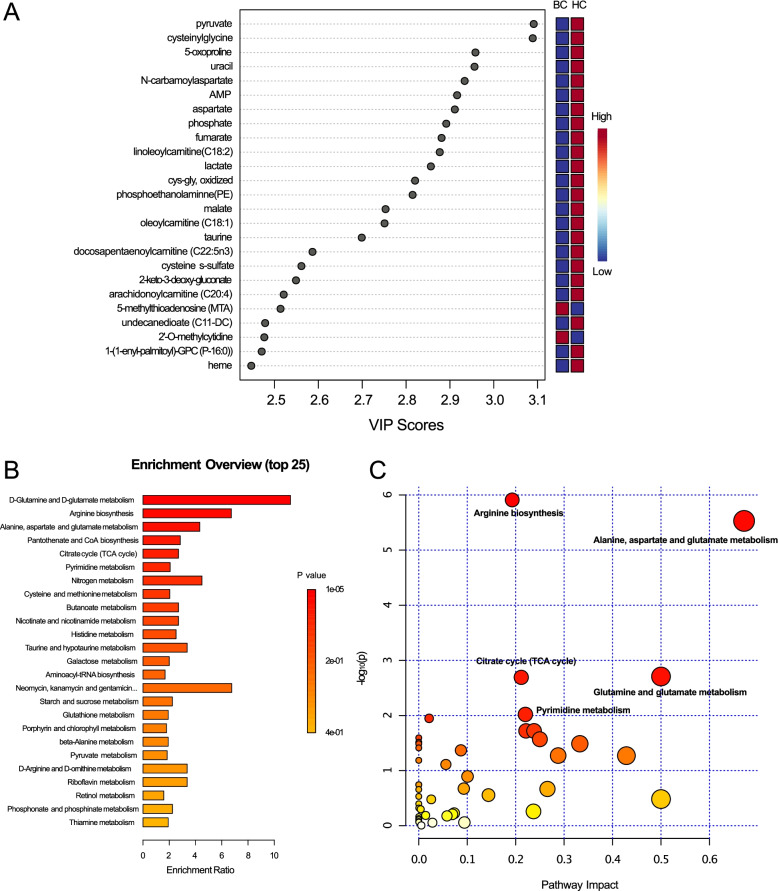
Differential metabolites and pathways between BC and HC groups. **A** Important metabolites identified by variable importance in projection (VIP) score obtained from OPLS-DA model. **B** Enrichment analysis results of the differential metabolites. Each column represents a metabolic pathway, the length of the column indicates the enrichment ratio. **C** Top significant functional pathways involved according to the deferentially expressed metabolites. Each circle represents a metabolic pathway, the larger the circle, the greater the pathway impact

Then, OPLS-DA was performed to evaluate the separation between groups and meanwhile identify the metabolites important for classification and discrimination between pairwise comparisons, and 100 permutation tests were conducted to validate the model. The results indicated an obvious separation between BC and HC groups (PC1 = 9.1%, PC2 = 13.8%) (Fig. 1D), similar to the benign and HC groups (PC1 = 14.1%, PC2 = 12%) (Fig. 1E). Both the two models obtained good values of explained ability (*R*_2_) and predictive ability (*Q*_2_) (*R*_2_*Y* = 0.960, *P* < 0.01 and *Q*_2_ = 0.892, *P* < 0.01; *R*_2_*Y* = 0.966, *P* < 0.01 and *Q*_2_ = 0.93, *P* < 0.01, respectively). However, the BC and benign groups were not completely separated, and the *Q*_2_ and *R*_2_ results were also bad (*R*_2_*Y* = 0.537, *P =* 0.83 and *Q*_2_ = 0.0344, *P* < 0.01) (Supplementary Fig. S2B). The above results indicated differences in the metabolic profiles between BC and HC, as well as between benign and HC. The results also showed that both BC and benign could be distinguished from HC by specific metabolite signatures. However, the current findings did not prove that metabolites could differentiate BC from benign. Therefore, we further analyzed the metabolic profiles between BC and HC. According to the OPLS-DA model, we identified 194 important metabolites (VIP > 1). Among the top 25 metabolites, the levels of pyruvate, cysteinylglycine, 5-oxoproline, uracil, *N*-carbamoylaspartate, AMP, aspartate, phosphate, and others were increased in HC. While the levels of 5-methylthioadenosine (MTA) and 2’-O-methylcytidine were increased in BC participants (Fig. 2A). The FC, VIP, and *P* values of each metabolite in the BC vs*.* HC comparison were shown in Supplementary Table 2.

### Differential metabolites and pathways between BC and HC groups

**Fig. 3:**
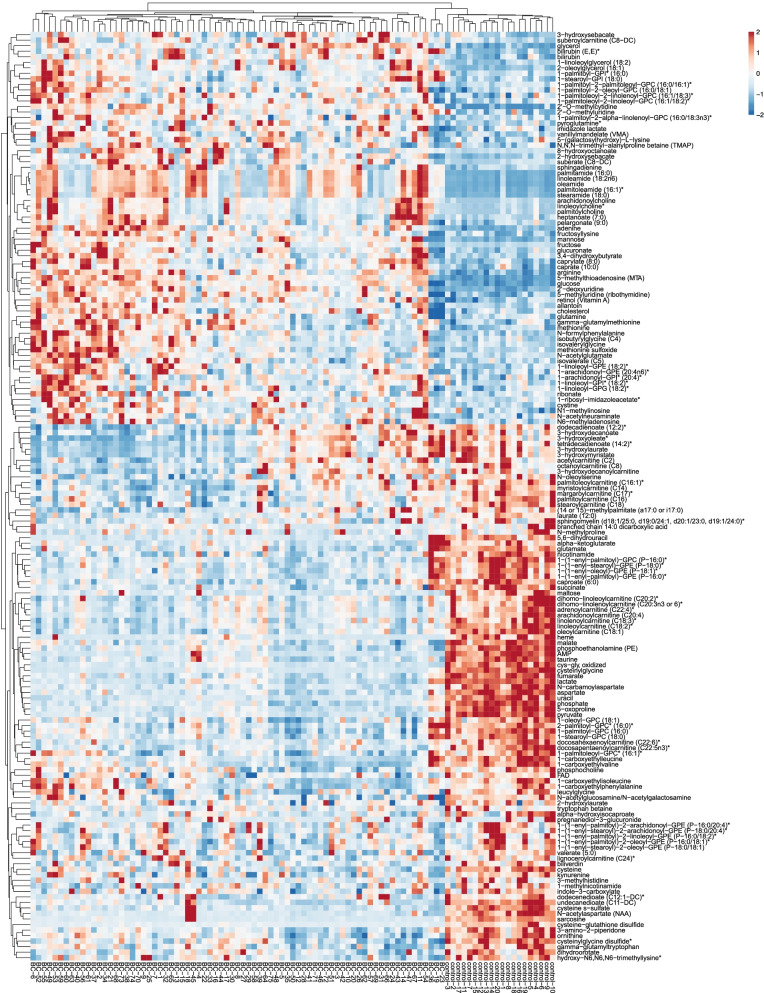
Hierarchical clustering analysis (HCA) of the differential metabolites between BC and HC groups. The colors from red to blue represent the relative levels of the metabolites between the two groups

A total of 187 significant metabolites were identified based on VIP > 1, FC >1.2 or <5/6, and *P* < 0.05. Among these metabolites, 17 metabolites were xenobiotics and 2 metabolites belonged to partially characterized molecules. The remaining 168 metabolites were further analyzed (Supplementary Table 3). Then, we performed pathway and enrichment analyses based on the 168 metabolites. According to the results of enrichment analysis (Fig. 2B), these significant metabolites were mainly concentrated in 46 metabolic pathways, including the glutamine and glutamate metabolic pathways (5 hits); arginine biosynthesis pathway (7 hits); alanine, aspartate, and glutamate metabolic pathways (9 hits); pantothenate and CoA biosynthesis pathway (4 hits); citrate cycle (TCA cycle) pathway (4 hits); and pyrimidine metabolism pathway (6 hits). In pathway analysis, we generated bubble plots to identify the specific metabolic pathways closely associated with BC. According to the -log10(*P*) value and pathway impact score two indicators, alanine, aspartate, and glutamate metabolism; glutamine and glutamate metabolism; arginine biosynthesis; citrate cycle (TCA cycle); and pyrimidine metabolism were the most important metabolic pathways (Fig. 2C). Hierarchical clustering (HCA) analysis demonstrated that the 168 differentially expressed metabolites could obviously separate BC from HC. Among them, 71 metabolites were higher in BC patients, while the remaining 97 metabolites showed an opposite pattern with a higher level in HC (Fig. 3).

### Proteomic profiles and integrative analysis with metabolomics

To understand the specific proteomic changes associated with the onset and progression of BC, we performed a global-scale plasma proteomics analysis. For the BC and HC groups, 9 plasma samples each were randomly selected form the training cohort. Then, 9 HC plasma samples were mixed into one loading sample. A total of 2103 proteins encoded by 1538 genes were identified, of which 1934 proteins encoded by 1407 genes were quantified. According to the criteria of 1.25-fold (FC > 1.25 or < 0.8), unique peptides ≥ 2, and peptide spectrum matches ≥ 5, 29 upregulated proteins and 2 downregulated proteins were observed in the BC group, which could be used as potential biomarkers for further evaluation and validation experiments (Supplementary Table 4). Gene Ontology (GO) analysis showed that these differential proteins had many important biological functions, including platelet degranulation, muscle filament sliding, and actin-myosin filament sliding. Protein–protein interaction network (PPI) analysis revealed that these proteins were closely related, the most closely related being transthyretin (TTR), carboxypeptidase B2 (CBP2), and vitamin D-binding protein (GC) (Supplementary Fig. S3A).

**Fig. 4:**
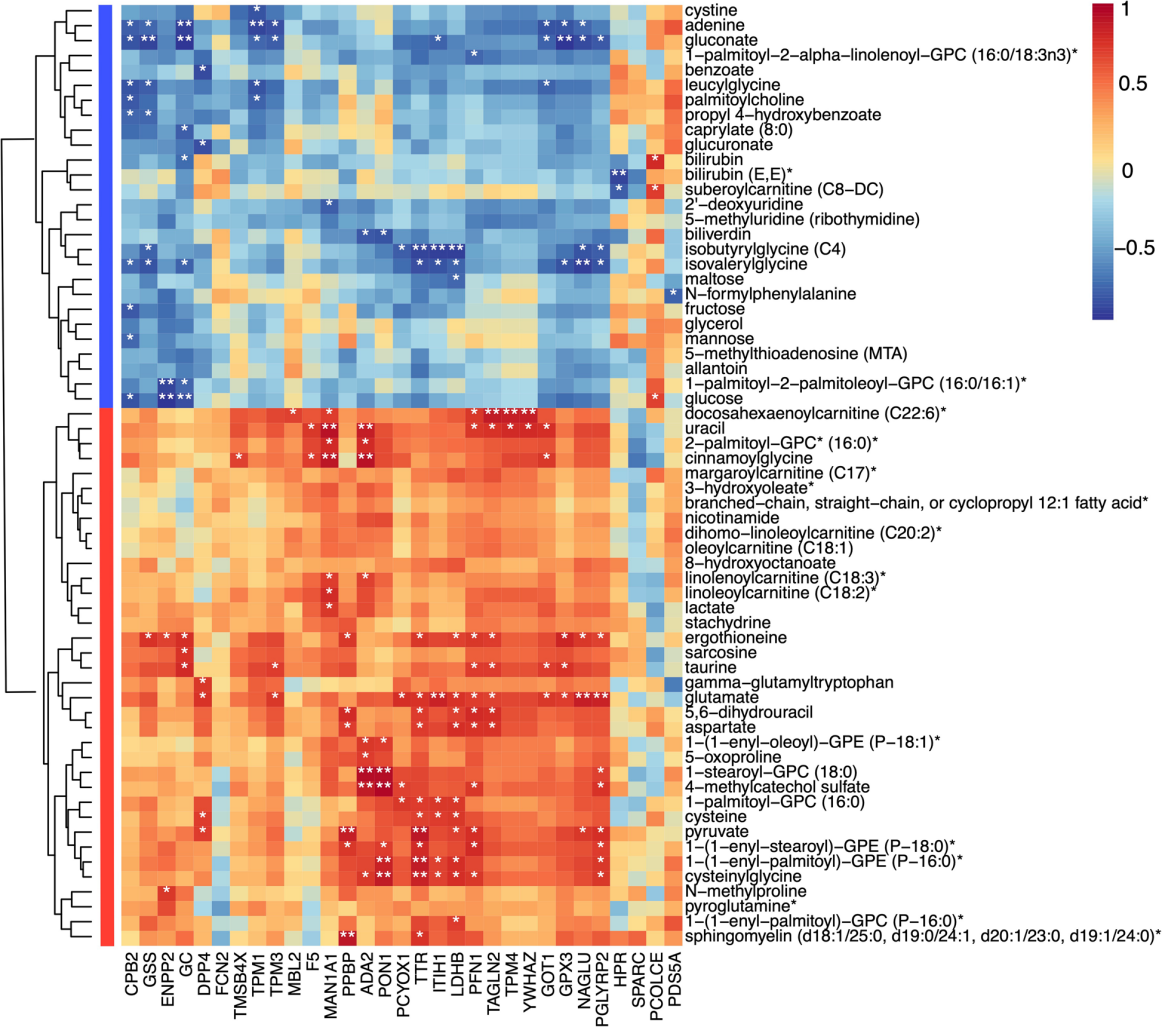
Heatmap of Spearman’s rank correlation analysis between differential metabolites and proteins. Blue: negative correlation; Red: positive correlation. Significant correlations regions were marked by stars (*P < 0.05, **P < 0.01)

**Fig. 5:**
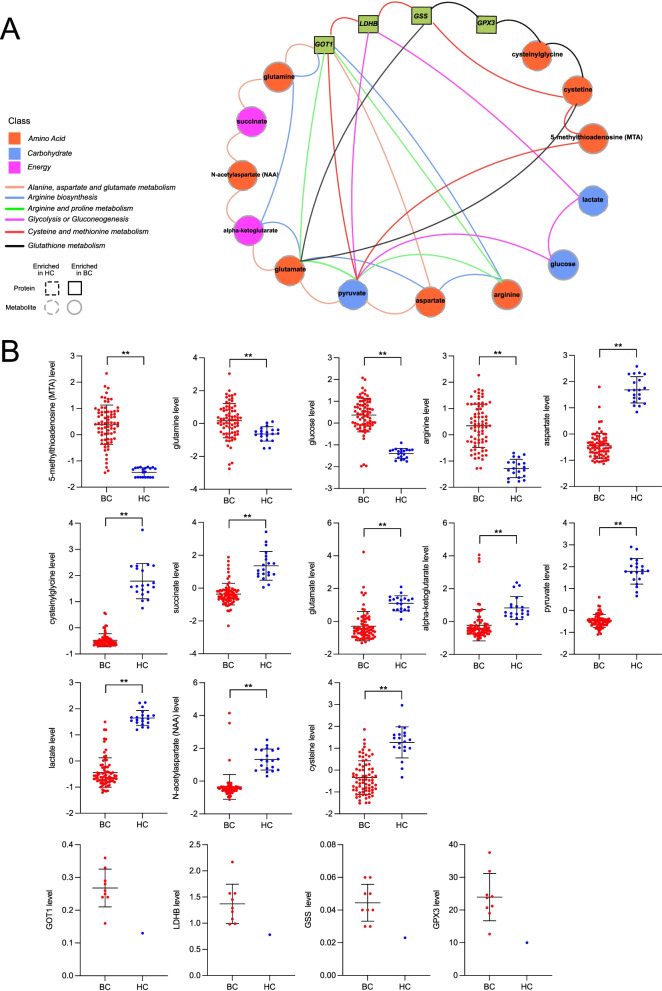
Integrated analysis of metabolomics and proteomics. **A** Joint pathway analysis of differential metabolites and proteins. Metabolites and proteins were presented in circles and squares; proteins and metabolites enriched in BC patients and those with HC individuals were indicated by solid lines and dashed lines, respectively. Metabolites and proteins involved in the same metabolic pathways were connected by lines, and each pathway had a corresponding color. **B** Differential expression of those metabolites and proteins involved in these metabolic pathways between BC and HC groups. **P < 0.01

We next carried out an integrative analysis to assess the connection between metabolites and proteins in BC patients. We first analyzed the correlation between the expression of differential metabolites and proteins. Differential metabolites were grouped into four clusters based on correlation values with proteins. The upper 2 clusters of metabolites were negatively correlated with these proteins, while the bottom 2 clusters of metabolites were positively correlated (Supplementary Fig. S3B). Specifically, the levels of fructose, mannose, and galactose metabolic pathway compounds (fructose and mannose); hemoglobin and porphyrin metabolic pathway compounds (bilirubin and biliverdin); and leucine, isoleucine, and valine metabolic pathway compounds (isobutyrylglycine) were negatively associated with these differential proteins. While levels of glutamate metabolic pathway compounds (glutamate and pyroglutamine); glutathione metabolic pathway compounds (5−oxoproline and cysteinylglycine); glycolysis, gluconeogenesis, and pyruvate metabolic pathway compounds (lactate and pyruvate); as well as methionine, cysteine, SAM, and taurine metabolic pathway compounds (taurine and cysteine) were positively associated with these proteins (Fig. 4). Then, we integrated the top biomarkers from metabolomics and proteomics data in a joint pathway analysis (Fig. 5A). This led to the identification of several important pathways which closely participated in the pathophysiologic processes of BC, including alanine, aspartate, and glutamate metabolism; arginine biosynthesis; arginine and proline metabolism; glycolysis or gluconeogenesis; cysteine and methionine metabolism; and glutathione metabolism. These metabolic pathways contained 13 key metabolites (5-methylthioadenosine (MTA), glutamine, glucose, arginine, aspartate, cysteinylglycine, succinate, glutamate, alpha-ketoglutarate, pyruvate, lactate, *N*-acetylaspartate (NAA), cysteine) and 4 important proteins (aspartate aminotransferase (GOT1), l-lactate dehydrogenase B chain (LDHB), glutathione synthetase (GSS), and glutathione peroxidase 3 (GPX3)) (Fig. 5B).

### Identification of plasma metabolic signatures for breast cancer diagnosis

In order to identify metabolites that could be used for early diagnosis of BC, machine learning algorithms, namely the random forest (RF) and support vector machine (SVM) models, were employed. For clinical use, the primary goal of breast cancer screening was to identify patients with breast cancer from populations. To do this, we first selected important metabolites between the BC and non-BC (benign + HC) groups based on the following criteria: *P* < 0.05, VIP > 0.5, and AUC > 0.6. This resulted in the identification of 428 metabolites. Then, we performed Lasso regression 10-fold cross-validation and random forest to further screen the metabolite biomarkers. Lasso regression identified 13 metabolites (Supplementary Fig. S4), and Random Forest identified 46 metabolites. 47 metabolites were finally selected after combing the 13 metabolites and 46 metabolites (Supplementary Table 5). The pathway analyses based on the 47 selected metabolites demonstrated the most important metabolic pathways were the glutamine and glutamate metabolism, alanine, aspartate and glutamate metabolism, arginine biosynthesis, and citrate cycle (TCA cycle) (Supplementary Fig. S5), which were consistent with the above differential metabolites participated pathways. And this emphasized that these metabolic pathways may play a critical role in the pathogennesis of breast cancer. To verify the values of the 47 identified metabolites in predicting BC, we used these 47 metabolites to train RF and SVM models.

**Fig. 6:**
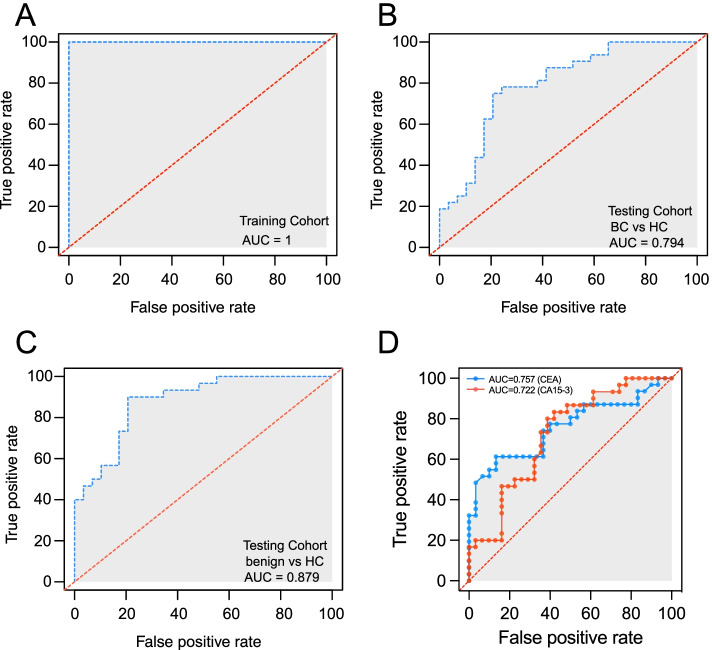
Specific metabolic signature-based diagnostic biomarkers for BC. **A** ROC curves of the prediction efficacy for the metabolites-based predictors in training cohort using SVM (AUC = 1). **B**, **C** The validation for the performance of the prediction model in testing cohort using SVM, BC vs. HC (AUC = 0.794) (**B**), benign vs. HC (AUC = 0.879) (**C**). **D** ROC curves of the prediction efficacy of the CA15-3 (AUC = 0.722) and CEA (AUC = 0.757)

Both RF (Supplementary Fig. S6A) and SVM (Fig. 6A) models were highly accurate in their prediction of BC in the training cohort (AUC = 0.998 and 1, respectively). Besides the training cohort, we measured the expression of metabolites in the internal testing cohort. The SVM model trained from the training cohort was applied to the testing cohort to further evaluate the model’s performance, and we found a good predictive power between BC and non-BC (AUC = 0.610) (Supplementary Fig. S6B). Besides the good performance of the model in distinguishing BC from non-BC, we further evaluated the performance in different subgroups of the testing cohort. For BC vs. HC, the AUC was 0.794 (Fig. 6B), and for benign vs. HC, the AUC was 0.879 (Fig. 6C). Both were higher than the two commonly used tumor markers for BC, CA15-3, and CEA (AUC = 0.722 and 0.757, respectively) (Fig. 6D). But the AUC was low for BC vs. benign (Supplementary Fig. S6C). These results indicated that the SVM model trained by 47 metabolites could effectively distinguish BC from HC individuals.

## Discussion

Early detection, diagnosis, and intervention of BC are challenging but crucial [22]. At present, the most commonly used BC screening methods include mammography and breast ultrasonography [9]. However, almost all breast nodules found in the above examinations usually need further scrutiny with breast biopsy to determine the nature of the nodules. This calls for the development of non-invasive and reliable plasma biomarkers which could be used to build cost-effective assays for routine clinical screening and thus substantially improve the management of BC.

BC is highly heterogeneous, and this heterogeneity is difficult to show by routine histopathological examination [23]. Cancer cells, however, often show obvious changes in cell metabolism, and such changes have shown a biochemical basis for tumorigenicity and malignancy [24]. Facilitated by the progress in technological and societal development, metabolomics has already been widely used in the clinic to help in the diagnosis of diseases [25]. Metabolomics is a new discipline involving the simultaneous qualitative and quantitative analysis of all metabolites in a given organism or cell during a given physiological period [26]. Unlike genomics and proteomics, metabolomics mainly reflects the end products of the cellular metabolic process, which, beyond the genome and proteome, represent the most downstream stage of vital movement [13]. The levels of these metabolites can be considered the final response of an organism to genetic or environmental changes, thus becoming an accurate reflection of disease phenotype [12]. Accordingly, metabolomics has been a pivotal tool for disease-biomarker identification and also a technique for discovering drivers of biological processes. Herein, we applied this approach to explore the specific metabolic signatures for specific use in BC diagnosis.

In this study, we performed metabolomics on plasma from 216 HC, benign, and BC subjects from two cohorts. In the training cohort, metabolomics analysis identified and quantified 917 metabolites. Obvious separation could be observed between BC and HC, as well as between benign and HC, indicating the existence of a specific metabolic profile for each condition. However, the separation between BC and benign was narrower. Similar group separation has been previously reported [27]. For BC vs. HC, our metabolomics results identified glutamate and glutamine metabolism, as well as alanine, aspartate, and glutamate and arginine biosynthesis metabolism were the most important pathways in BC, suggesting extensive metabolic disorders during BC progression. Previous study has demonstrated that the alanine, aspartate, and glutamate pathway was a critical biological pathway for early diagnosis of BC [28]. Arginine metabolic pathway has been identified as potential therapeutic targets and diagnostic biomarkers for gastric cancer [29]. Most metabolites in these three metabolic pathways were downregulated in BC patients compared to HC, suggesting that these three metabolic pathways and metabolites in these metabolic pathways may play an important role in the pathophysiological processes of BC. However, a defining link between these metabolic pathways and BC development still needs further clarification.

In fact, proteins and metabolites interact. On the one hand, proteins can affect metabolite signatures, whereas, conversely, metabolites can affect the level of proteins through enzymatic reaction [30, 31]. Thus, the combined analysis of proteomics and metabolomics can provide us with a more comprehensive understanding of BC. The proteomics data specifically identified 29 upregulated proteins, such as GOT1, LDHB, GPX3, and GSS, and 2 downregulated proteins, including dipeptidyl peptidase 4 (DPP4) and GC) in BC. Among the 31 differential proteins, GOT1, LDHB, GPX3, and GSS were closely connected with metabolites and collectively participated in several important metabolic pathways, including alanine, aspartate and glutamate pathway, arginine biosynthesis pathway, arginine and proline metabolism, glycolysis or gluconeogenesis, cysteine and methionine metabolism, and glutathione metabolism.

What are the mechanisms and functions of these four proteins in BC patients? GOT1 is a transaminase mainly existed in cardiomyocytes and the mitochondria of hepatocytes. It is well known that GOT1 levels increase is usually a response to injury in hepatocytes and cardiomyocytes. GOT1 catalyzes the production of pyruvate and glutamate from alanine and ketoglutarate and is therefore closely related to the citrate cycle and the metabolic pathways involved in glutamate. In recent years, the GOT1 metabolic pathway has been found to play an important role in many cancers, such as glioblastoma multiforme, small cell lung cancer, pancreatic ductal adenocarcinoma (PDCA), and BC [32–34]. The effects of GOT1 on cancer cells have been fully demonstrated in PDAC. Specifically, the inhibition of GOT1 activity could suppress the growth of PDAC cells. Meanwhile, GOT1 could regulate the balance between energy metabolism and reactive oxygen species (ROS) in acidosis [35]. Therefore, GOT1 knockdown could disrupt nucleotide metabolism, glycolysis, and redox homeostasis in PDAC cells [36]. Besides, GOT1 knockdown also could accelerate pancreatic cancer cell death by regulating iron metabolism and ferroptosis [37]. LDHB is one of the important enzymes of glycolysis and gluconeogenesis, typically used to monitor myocardial infarction. LDHB plays an important role in the reciprocal transformation of pyruvate and lactic acid, so it is crucial to the cancer-specific Warburg effect, and thus, it may be an important cancer-related target. Studies found that LDHB could be regulated by the kruppel-like factor 14 (KLF14) transcription factor to regulate glycolysis [38] and be regulated by the fibroblast growth factor receptor 1 (FGFR1) to regulate Warburg effect [39]. The increased LDHB promoted more pyruvate comfort to lactic acid, and the increased lactate levels in the tumor microenvironment could promote tumor invasion and metastasis through the activation of vascular growth factor, and promotion the expression activity of hyaluronan with its receptor CD44 [40]. In addition, LDHB expression had different effects on different tumors, promoting or inhibiting tumor growth. Many studies have found that LDHB could promote the proliferation, migration, and invasion of cancer cells by regulating apoptosis and autophagy [41–43]. GPX3, a selenoprotein, can catalyze the reduction of hydrogen peroxide and other hyperoxides by reduced glutathione (GSH), so as to remove reactive oxygen species and reduce DNA damage. Studies have founded that GPX3 played a dual role in tumor development. On the one hand, it can act as a tumor suppressor protein, while on the other hand, it can also act as a pro-survival protein during the progression of tumor [44]. Specifically, the over-expression of GPX3 could decrease the clonogenic growth, xenograft tumor size, and migration and invasion of prostate cancer cells [45]. Additionally, GPX3 over-expression similarly suppressed the proliferation and metastasis of cancer cells [46]. The anti-tumor activity of GPX3 depends on its downregulation of the oxidant-regulated tumor-promoting signaling pathway. However, other studies found that GPX3 over-expression can promote tumor progression [47, 48]. Based on these findings, we speculate that GOT1, LDHB, and GPX3 may affect BC through the same mechanisms as those mentioned above, such as disrupting glycolysis and nucleotide metabolism, promoting ferroptosis, or downregulating the oxidant-regulated tumor-promoting signaling pathway. However, these hypotheses also need further research to verify.

To further identify the specific metabolic signatures for BC diagnosis, a SVM model was employed, and it defined a predictive model with 47 metabolites. Among these 47 metabolites, several metabolites were shown to be closely associated with cancer. Sphingomyelins (SM), most of which were upregulated in BC, are a main class of sphingolipids. SMs are the basic elements of the cell membrane and play a critical role in cellular function. The proliferative effects of SMs can be explained by several possible mechanisms. The metabolites of SMs, including ceramide (CER), sphingosine (SPH), and sphingosine-1-phosphate (S1P), as important signaling molecules, regulated many cellular life activities, including cellular proliferation, growth, apoptosis, and autophagy [49, 50]. Many studies have found the increased SMs were closely associated with a worse prognosis in ovarian, breast, prostate, and colorectal cancer [51, 52]. Additionally, SMs have been identified as diagnostic and prognostic biomarkers of several kinds of cancer, such as endometrial cancer and epithelial ovarian cancer [53, 54]. Glutamate, downregulated in BC, is a key excitatory neurotransmitters that participates in biosynthesis, metabolic, and carcinogenic signaling pathways [55]. Glutamate can combine with ammonia to form glutamine, then dissociated after being transported to the liver and kidney, which is an important way to protect against ammonia poisoning. In addition, glutamate is participated in the urea synthesis and nucleotide metabolism. Interestingly, the decreased glutamate in the plasma was conversely related to the increased glutamate in BC tissues [56], suggesting that BC cells absorbed large amounts of glutamate from blood circulation to maintain the life activities of tumor cells. The upregulation of glutamate has also been observed in other types of cancer, such as serous ovarian cancer and lung adenocarcinoma [57, 58]. Studies found that glutamate could accelerate tumorigenesis by activating the alpha-amino-3-hydroxy-5-methyl-4-isoxazolepropionic acid receptors (AMPAR), as well as promoted the invasion and migration of pancreatic cancer cells through activation of the MAPK pathway [59]. In BC, glutamate was also found to be important in the induction of HIF1α under normoxic conditions [60]. Cysteine, downregulated in BC, is a common amino acid in organisms. Similarly, the decreased cysteine in the plasma is conversely related to the increased cysteine in BC tissues [56], suggesting BC cells utilized more cysteine. Cysteine actively participates in cancer metabolic reconstruction in different ways. For instance, it can act as an ingredient in glutathione redox reaction. It is also a substrate for producing hydrogen sulphide (H2S) that stimulates cellular bioenergetics. Finally, it provides a carbon source for energy production and biomass production [61]. A cysteine-related metabolic pathway, cysteinyl leukotriene pathway (CysLT), is closely associated with cancer [62]. CysLT can promote the survival and proliferation of many cancer cells. However, its disruption reduced cell viability and led to cell death in many types of cancer cells, including breast cancer, lung cancer, and neurological malignancies. Moreover, CysLT was also related to chemoresistance of cancer, and its disruption could reverse chemoresistance. Based on the above findings, we believe that the panel of 47 metabolites, including SM, glutamate, and cysteine, closely participates in the pathophysiology of BC and that it can achieve high efficacy in the diagnosis of breast cancer. Of course, the specific mechanisms need further study.

Our study also has several limitations. First, the sample size was relatively small. All participants enrolled from one single center, and no external testing cohort was established. Second, the samples used for proteomics analysis were too small, and the results were not verified. Third, the exact mechanism underlying the involvement of these identified metabolites in BC is still not clear. Thus, further proteomics and metabolomics analysis with larger sample size from multiple centers is required to validate our results. A mechanistic study of several metabolites and proteins in the pathophysiology of breast cancer is the focus of our next study.

## Conclusions

In conclusion, we have characterized the systematic changes of plasma metabolome and proteinogram in BC patients. The alanine, aspartate and glutamate pathway, glutamine and glutamate metabolic pathway, and arginine biosynthesis pathway were the crucial metabolic pathways in the pathogenesis of breast cancer. We also identified a panel of 47 metabolites, including sphingomyelins, glutamate, and cysteine, which could be effectively used for BC diagnosis. Although it is too early to infer that these biomarkers will replace the current BC screening used in the clinic, our study did succeed in demonstrating that the analysis of plasma metabolomics provides a high confidence interval for exploring diagnostic biomarkers for BC. We believe that our findings can contribute to the development of effective diagnostic tools with extensive applications in the clinical screening of breast cancer after further experimental confirmation.

## Supplementary Information

**Additional file 1: Figure S1.** Details of the study design and research cohorts. **Figure S2.** Comparison of metabolomics between BC and benign groups. (A) Volcano plot showing the metabolites that were significantly different between BC and benign groups. Each point represents a metabolite, red: up-regulated metabolites, blue: down-regulated metabolites. (B) Score plots of OPLS-DA models between BC and benign groups. Each point represents a sample, red: BC patients, green: benign patients. **Figure S3.** Integrated analysis of metabolomics and proteomics. (A) Protein-protein interaction network (PPI) analysis of the differential proteins. (B) Heatmap of Spearman’s rank correlation analysis between differential metabolites and proteins. Blue: negative correlation; Red: positive correlation. Significant correlations regions were marked by stars (*P < 0.05, **P < 0.01). **Figure S4.** Features selection using the Lasso regression model using 10-fold cross-validation. Dashed vertical lines were drawn at the best values by using the minimum criteria and the 1 standard error of the minimum criteria (the 1-SE criteria). **Figure S5.** Top significant functional pathways involved according to the selected 47 candidate metabolites which used for building a diagnostic model for breast cancer. Each circle represents a metabolic pathway, the larger the circle, the greater the pathway impact. **Figure S6.** Specific metabolic signatures based diagnostic biomarkers for BC. (A) ROC curves of the prediction efficacy for the metabolites-based predictors in Training Cohort using RF (AUC = 0.998 ). (B-C) The validation for the performance of the prediction model in Testing Cohort using SVM, BC vs non-BC (AUC = 0.610) (B), BC vs benign (AUC = 0.453) (C).

**Additional file 2.**

## Data Availability

The raw mass spectrometry data generated and analyzed during the current study are not publicly available due to needs of our further study, but are available from the corresponding author on reasonable request.

## Abbreviations

BC: Breast cancer
HC: Healthy control
BRCA1/2: Breast cancer-associated genes 1 and 2
HER2: Human epidermal growth factor receptor 2
EGFR: Epidermal growth factor receptor
TM: Tumor markers
CEA: Carcinoembryonic antigen
CA15-3: Carbohydrate antigen 15-3
BMI: Body mass index
QC: Quality control
UPLC-MS/MS analysis: Ultrahigh Performance Liquid Chromatography-Tandem Mass Spectroscopy Ananlysis
FC: Fold change threshold
OPLS-DA: Orthogonal projections to latent structure discriminant analysis
VIP: Variable importance in the projection
GO analysis: Gene Ontology analysis
PPI: Protein–protein interaction network analysis
SVM: Support vector machine
RF: Random forest
ROC: Receiver operating characteristic curves
AUC: Area under the curve
PFPeA: Perfluoropentanoic acid
FA: Formic acid
GOT1: Aspartate aminotransferase
LDHB: l-lactate dehydrogenase B chain
GSS: Glutathione synthetase
GPX3: Glutathione peroxidase 3
TTR: Transthyretin
CBP2: Carboxypeptidase B2
GC: Vitamin D-binding protein
PDCA: Pancreatic ductal adenocarcinoma
ROS: Reactive oxygen species
KLF14: Kruppel-like factor 14
FGFR1: Fibroblast growth factor receptor 1
SM: Sphingomyelins
CER: Ceramide
SPH: Sphingosine
S1P: Sphingosine-1-phosphate
AMPAR: Alpha-amino-3-hydroxy-5-methyl-4-isoxazolepropionic acid receptors
CysLT: Cysteinyl leukotriene pathway
