# Supplementary material for: Integrative analysis of plasma metabolomics and proteomics reveals the metabolic landscape of breast cancer
Source: Cancer Metab. 2022 Aug 17;10:13. doi: 10.1186/s40170-022-00289-6 (PMC9382832; doi:10.1186/s40170-022-00289-6)
Supplement: Supplementary file 1 — Additional file 1: Figure S1. Details of the study design and research cohorts. Figure S2. Comparison of metabolomics between BC and benign groups. (A) Volcano plot showing the metabolites that were significantly different between BC and benign groups. Each point represents a metabolite, red: up-regulated metabolites, blue: down-regulated metabolites. (B) Score plots of OPLS-DA models between BC and benign groups. Each point represents a sample, red: BC patients, green: benign patients. Figure S3. Integrated analysis of metabolomics and proteomics. (A) Protein-protein interaction network (PPI) analysis of the differential proteins. (B) Heatmap of Spearman’s rank correlation analysis between differential metabolites and proteins. Blue: negative correlation; Red: positive correlation. Significant correlations regions were marked by stars (*P < 0.05, **P < 0.01). Figure S4. Features selection using the Lasso regression model using 10-fold cross-validation. Dashed vertical lines were drawn at the best values by using the minimum criteria and the 1 standard error of the minimum criteria (the 1-SE criteria). Figure S5. Top significant functional pathways involved according to the selected 47 candidate metabolites which used for building a diagnostic model for breast cancer. Each circle represents a metabolic pathway, the larger the circle, the greater the pathway impact. Figure S6. Specific metabolic signatures based diagnostic biomarkers for BC. (A) ROC curves of the prediction efficacy for the metabolites-based predictors in Training Cohort using RF (AUC = 0.998 ). (B-C) The validation for the performance of the prediction model in Testing Cohort using SVM, BC vs non-BC (AUC = 0.610) (B), BC vs benign (AUC = 0.453) (C). [file 40170_2022_289_MOESM1_ESM.docx]

**Supplementary Figures**

**
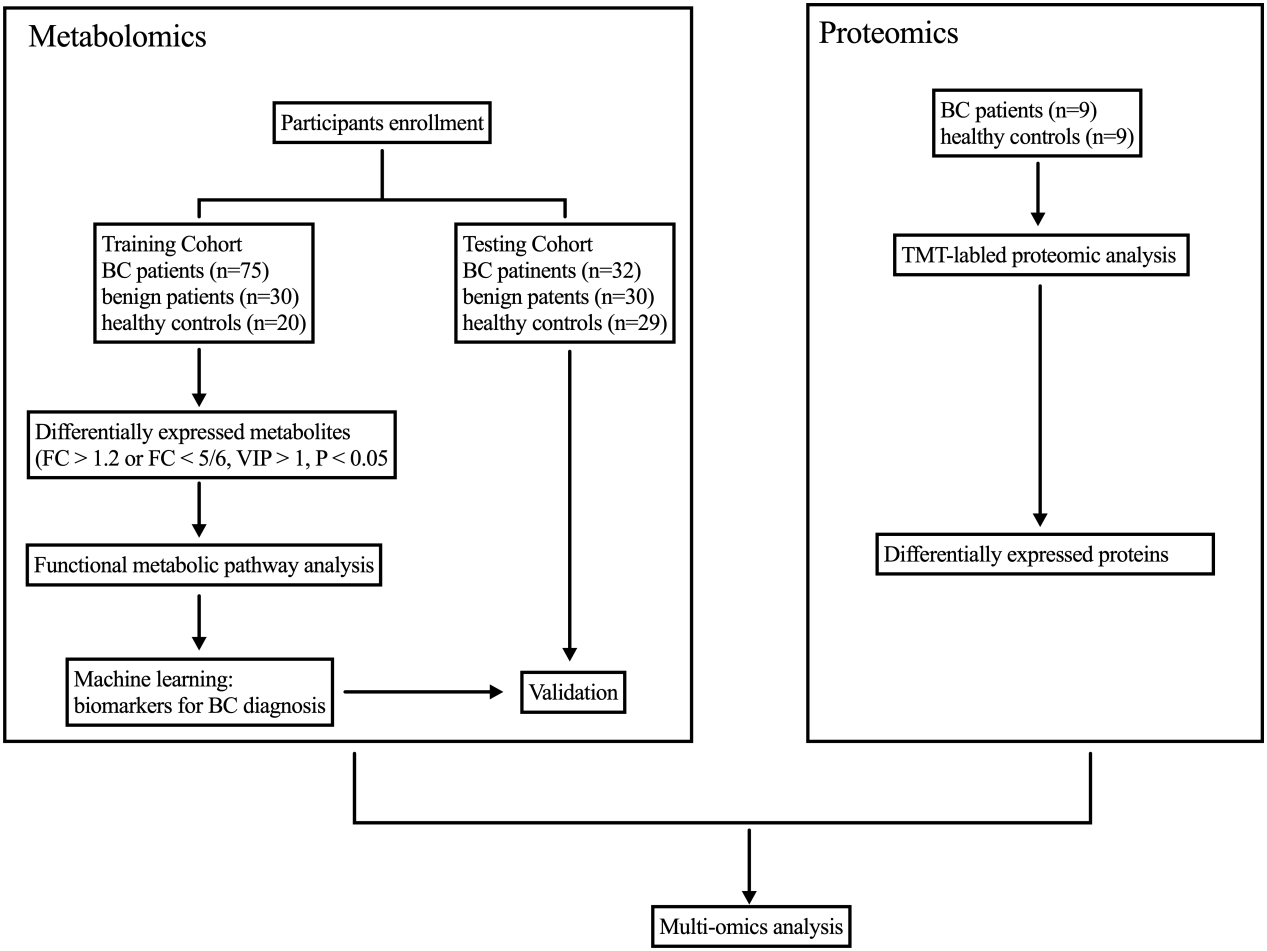
**

Figure S1. Details of the study design and research cohorts.


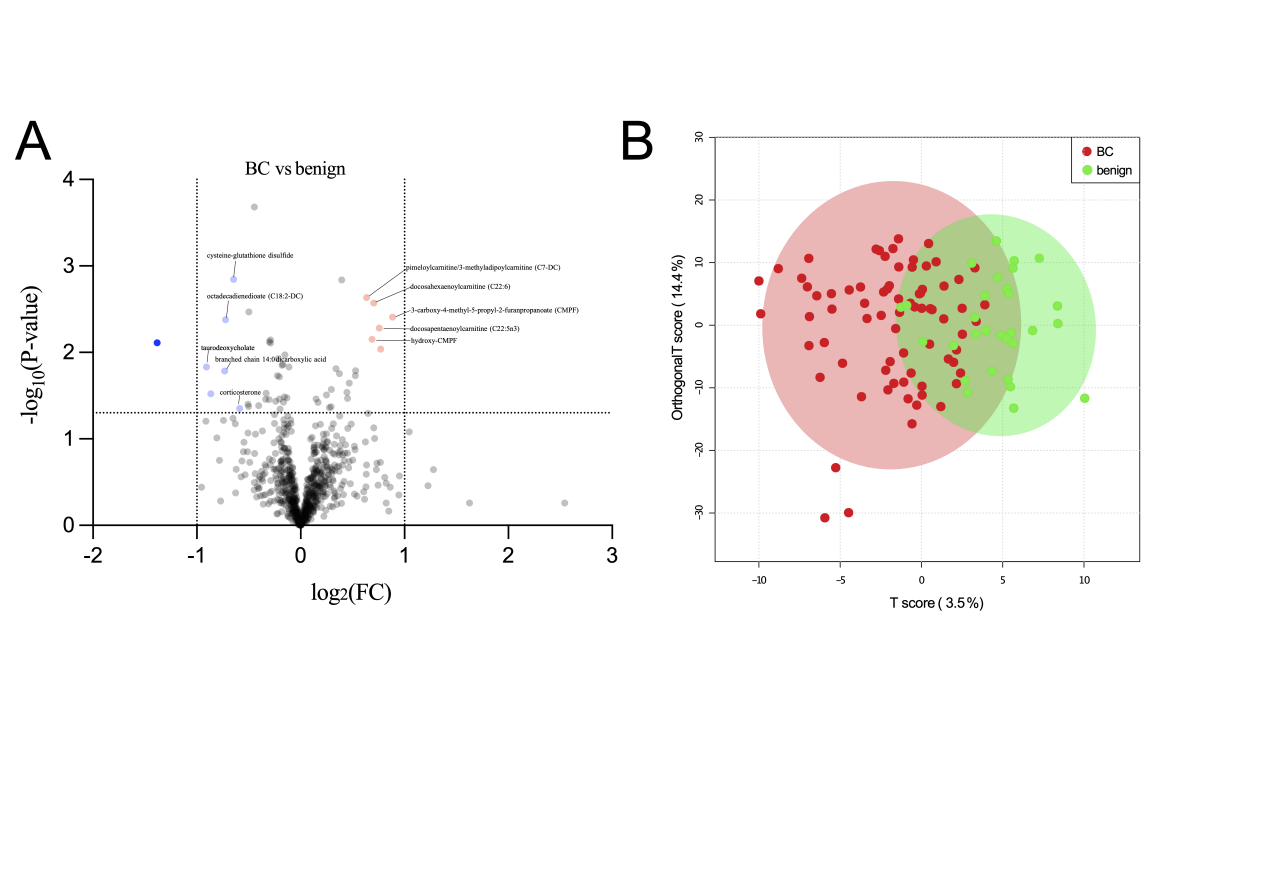


Figure S2. Comparison of metabolomics between BC and benign groups. (A) Volcano plot showing the metabolites that were significantly different between BC and benign groups. Each point represents a metabolite, red: up-regulated metabolites, blue: down-regulated metabolites. (B) Score plots of OPLS-DA models between BC and benign groups. Each point represents a sample, red: BC patients, green: benign patients.


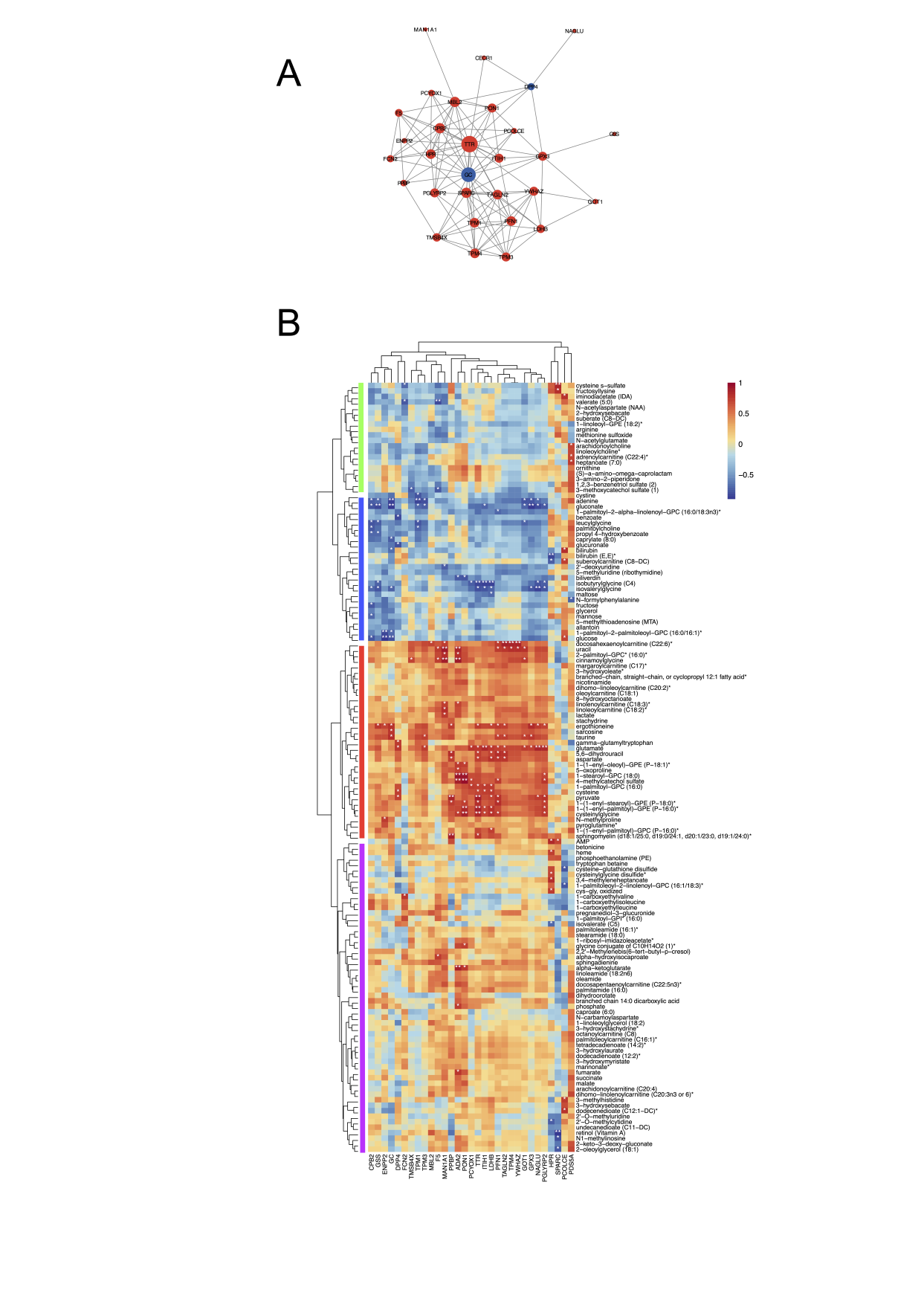


Figure S3. Integrated analysis of metabolomics and proteomics. (A) Protein-protein interaction network (PPI) analysis of the differential proteins. (B) Heatmap of Spearman’s rank correlation analysis between differential metabolites and proteins. Blue: negative correlation; Red: positive correlation. Significant correlations regions were marked by stars (**P* < 0.05, ***P* < 0.01).


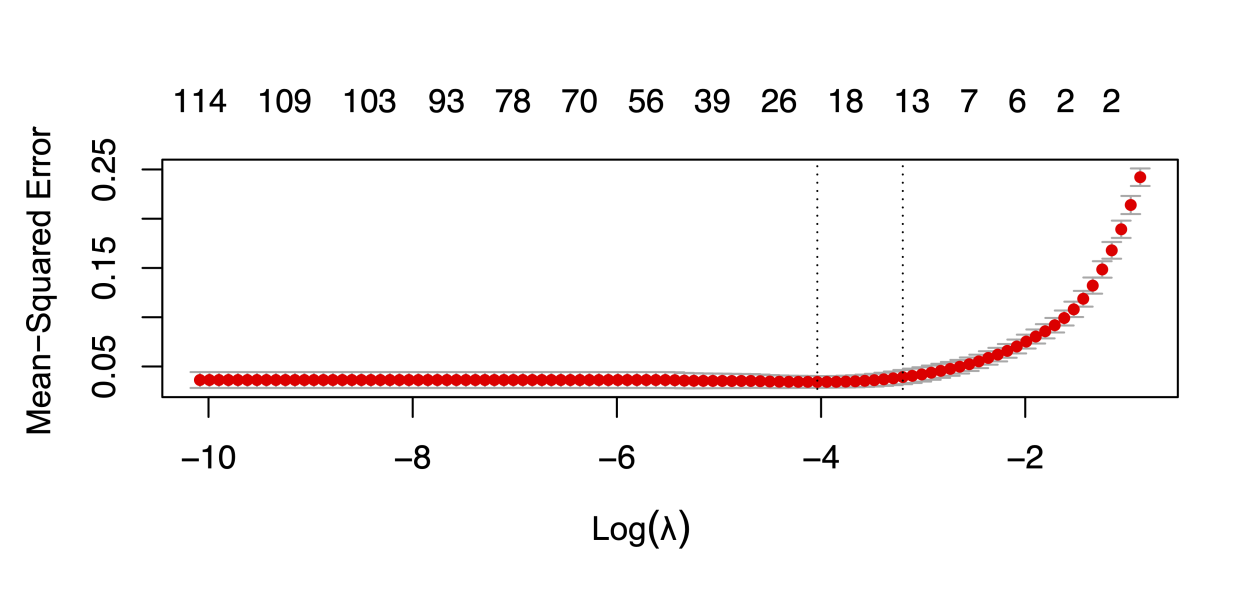


Figure S4. Features selection using the Lasso regression model using 10-fold cross-validation. Dashed vertical lines were drawn at the best values by using the minimum criteria and the 1 standard error of the minimum criteria (the 1-SE criteria).


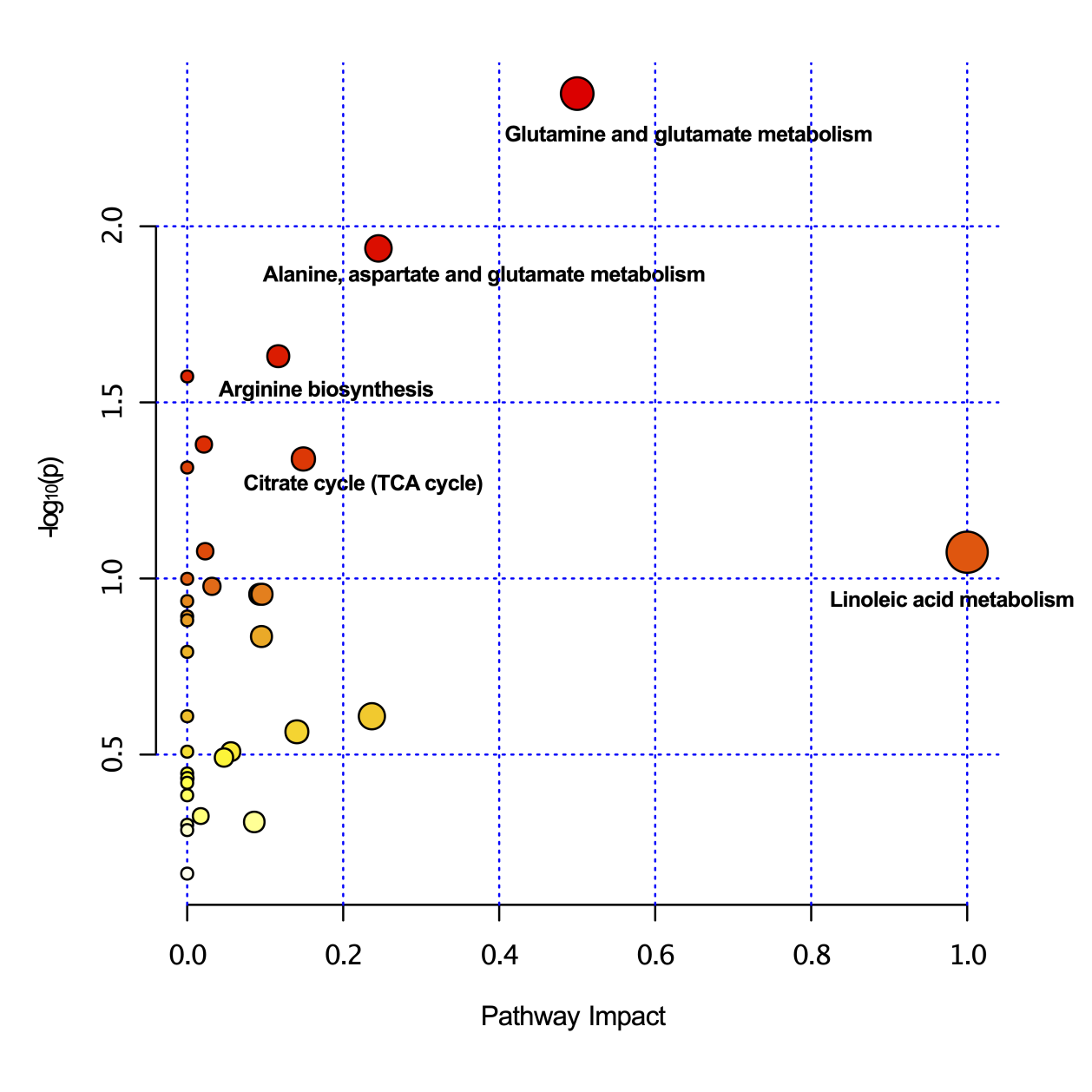


Figure S5. Top significant functional pathways involved according to the selected 47 candidate metabolites which used for building a diagnostic model for breast cancer. Each circle represents a metabolic pathway, the larger the circle, the greater the pathway impact.


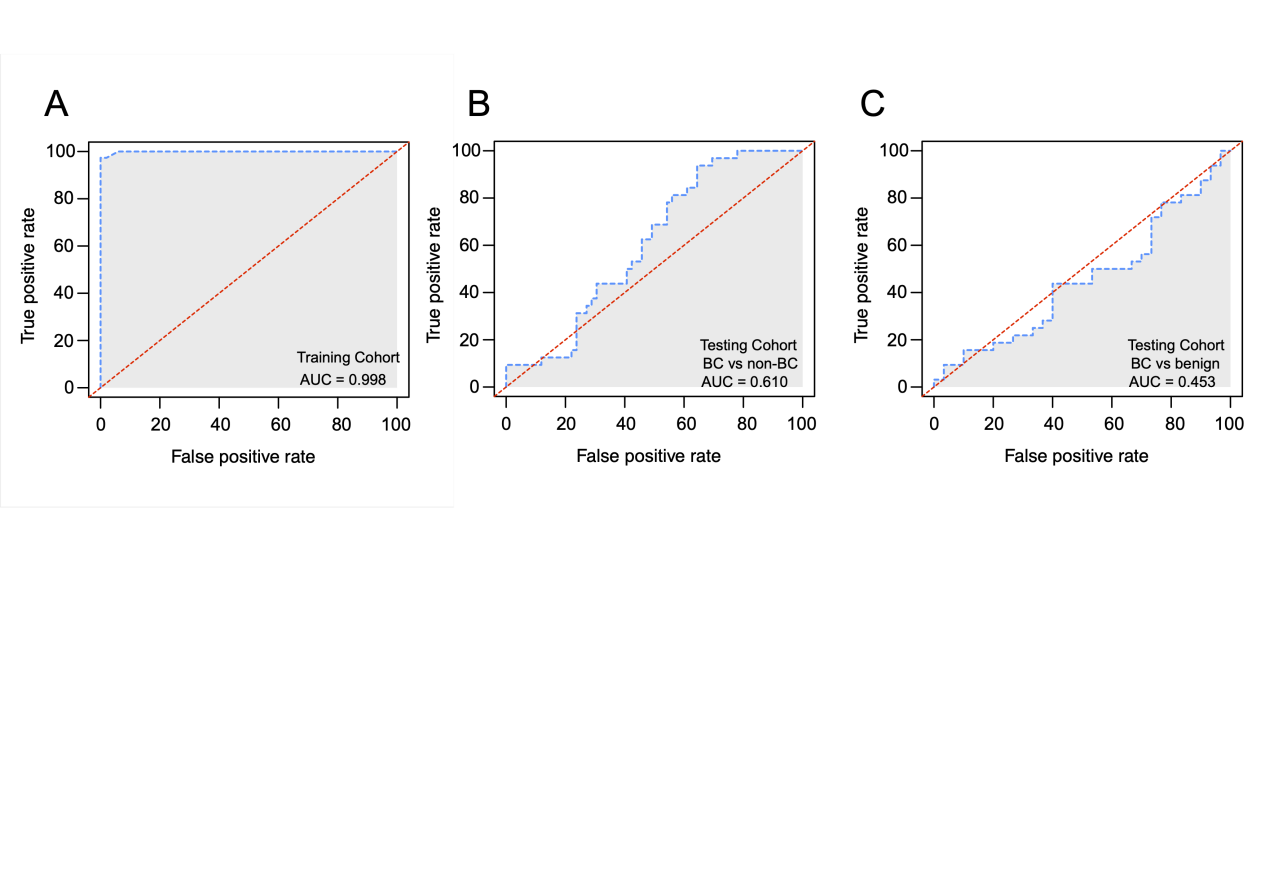


Figure S6. Specific metabolic signatures based diagnostic biomarkers for BC. (A) ROC curves of the prediction efficacy for the metabolites-based predictors in Training Cohort using RF (AUC = 0.998 ). (B-C) The validation for the performance of the prediction model in Testing Cohort using SVM, BC vs non-BC (AUC = 0.610) (B), BC vs benign (AUC = 0.453) (C).
